# Editorial: Self-understanding and other-understanding in personality pathology

**DOI:** 10.3389/fpsyt.2024.1328860

**Published:** 2024-01-19

**Authors:** Majse Lind, Espen Jan Folmo, Erin A. Kaufman

**Affiliations:** ^1^ Department of Communication and Psychology, Aalborg University, Aalborg, Denmark; ^2^ INSEAD, Fontainebleau, France; ^3^ Department of Psychiatry, University of Utah, Salt Lake City, UT, United States

**Keywords:** IDC-11, self-understanding, other-understanding, personality disorder (PD), Personality pathology in adolescence, dimensional models, AMPD

Personality disorders (PD) are serious mental illnesses characterized by rigid patterns of dysfunctional thinking, feeling, and behaving within several life domains (1). PD research and classification have begun shifting away from categorical approaches toward more dimensional conceptualizations (e.g., the alternative model presented in DSM-5 Section III and ICD-11). Such models focus on quantifying the *degree* of personality pathology across key dimensions (1, 2). Within the alternate model, dysfunction in self-understanding (i.e., identity, self-direction) and other-understanding (i.e., empathy, intimacy) are presented as central building blocks to PD pathology. Persons are evaluated on a continuum from no personality dysfunction to severe dysfunction in these areas (i.e., in need of treatment; 3). Given the centrality of self- and other understanding in novel models of PD, we devoted this Research Topic to expanding our understanding of *why*, *how*, and *when* self-understanding and other-understanding are critical to PD emergence and maintenance.

Regarding *why*, we sought to include empirical and conceptual papers examining the incremental validity of self-understanding and other-understanding in explaining personality pathology. Cervantes et al., highlighted how self-understanding and other-understanding in PD have seldomly been internally evaluated. The authors found an association between intimacy and borderline PD features mediated by identity functioning among adolescents highlighting self-understanding as a likely driver in PD. Somewhat counterintuitively, extant research shows that individuals with PD perform well in other-understanding tasks despite reporting severe interpersonal difficulties. Indeed, Grealy et al. found that individuals with more borderline PD features performed better than individuals with fewer PD features when identifying negative valence stimuli. Finally, we also include papers that emphasize the importance of self-other understanding as treatment process and/or outcome. Two studies used a qualitative approach to explore patient and therapist attitudes about a short-term mentalization-based therapy (MBT). MBT is an evidence-based treatment focused on fostering self-other understanding (or mentalizing) as a means of recovery (4). Central themes emerged indicating both benefits and shortfalls with short-term treatment. Relatedly, Marconi et al. showed promising results of a Metacognitive Interpersonal Therapy-informed treatment (5) in an outpatient sample. Pol et al. examined changes in narrative identity among individuals with PD and found increased agency pre- to post-treatment (i.e., Dialectical Behavioral Therapy, DBT; 6 & Schema-Focused Therapy; 7), indicating a strengthened sense of authorship and authority in one’s life story as crucial for recovery (see also 8). Supporting these findings, Timberlake and Fesel presented a case study of a patient with avoidant PD before, immediately after, and six months following short-term psychodynamic psychotherapy (STPP: 9) termination. Changes in narrative identity were assessed based on therapy transcripts and life narrative interviews. The authors found increased agency and coherence following psychotherapy. Communion fulfillment decreased during therapy but then rose post-treatment, indicating that interpersonal difficulties may need attention before PD amelioration. Lastly, through qualitative analyses, Tobiassen et al. found persons with PD crafted narratives about therapeutic change coinciding with the specific treatment they had received. That is, patients receiving DBT (6) highlighted learning tools and techniques, whereas patients receiving MBT (4) emphasized exploration to create procedural learning.

In terms of *how*, we sought articles that would articulate innovative methods for assessing self-other understanding in the context of personality pathology (e.g., state-of-the-art designs, newly developed measures). Juul highlighted several methodological limitations related to the quality of randomized clinical trials targeting PD and offered several recommendations for future research. Howard presented a conceptual paper on the importance of bridging the gap between typical and pathological personality development. He argued that PD may be organized by deficits in *self as identity* and *self as socially interdependent*. This Research Topic also gives voice to novel aspects of self-other understanding in a PD context. Lind emphasized the importance of narrative identity, or the internal and dynamic story of a person’s past, present, and presumed future (10). Narrative identity is a temporal understanding of the self and is largely overlooked in PD research - particularly the maladaptive narrative ecology in which the person coexists. Dr Lind emphasized the importance of taking the narrative ecology into account when studying the person with PD.

In terms of *when*, it has long been acknowledged that PD diagnoses do not emerge without developmental precursors. However, the appropriateness of diagnosis among adolescence has been questioned historically, given the wax and wane of PD symptoms and difficulties in identifying *which* adolescents will continue on a PD trajectory into adulthood (11, 12). Evidence indicates PD can be reliably identified in early adolescence (13, 14) and instruments show comparable reliability to those used with adults (15). In a dimensional model, there is no fixed age limit for diagnosis. Instead, a lifespan approach applies such that symptoms are interpreted in the individual’s developmental context (see also 16). A dimensional approach to diagnosis creates opportunity for researchers and clinicians to re-focus on PD among adolescence. Yet, we have little knowledge regarding valid predictors or the underlying causes of PD using “real world data” outside a clinical context. Bogaerts et al. examined developmental trajectories related to identity among a large sample of Finish adolescents and how these related to borderline PD and other personality characteristics. They showed that maladaptive identity trajectories were associated with PD and may signify a risk-factor for later disorder onset (but also a window of prevention).

The Research Topic was devoted to cementing the crucial role of self-understanding and other-understanding in PD. After PD onset, self-other understanding can be used as a therapeutic tool generating change in PD and as an important therapeutic outcome reflecting such change. In tandem, other papers illustrated how spotting maladaptive developmental trajectories in self-other understanding early in development can help prevent PD from “growing up”. We would like to thank all authors for their valuable contributions to the special issue.

## Author contributions

ML: Writing – original draft. EF: Writing – review & editing. EK: Writing – review & editing.
